# Ovine Hair Follicle Stem Cells Derived from Single Vibrissae Reconstitute Haired Skin

**DOI:** 10.3390/ijms160817779

**Published:** 2015-08-03

**Authors:** Huishan Zhang, Shoubing Zhang, Huashan Zhao, Jingqiao Qiao, Shuang Liu, Zhili Deng, Xiaohua Lei, Lina Ning, Yujing Cao, Yong Zhao, Enkui Duan

**Affiliations:** 1 State Key Laboratory of Stem Cell and Reproductive Biology, Institute of Zoology, Chinese Academy of Sciences, Beijing 100101, China; E-Mails: zhanghs@ioz.ac.cn (Hui.Z.); zhaohuashan@gmail.com (Hua.Z.); qiaojingqiao732@163.com (J.Q.); liushuang@ioz.ac.cn (S.L.); dengzhili@ioz.ac.cn (Z.D.); leixh@ioz.ac.cn (X.L.); ningln@ioz.ac.cn (L.N.); caoyj@ioz.ac.cn (Y.C.); 2 Department of Histology & Embryology, School of Basic Medical Sciences, Anhui Medical University, Hefei 230032, China; E-Mail: sdrzzhang@163.com; 3 University of Chinese Academy of Sciences, Beijing 100049, China; 4 State Key Laboratory of Membrane Biology, Institute of Zoology, Chinese Academy of Sciences, Beijing 100101, China; E-Mail: zhaoy@ioz.ac.cn

**Keywords:** hair follicle stem cells, keratinocytes, sheep, microdissection, organ culture, skin reconstitution

## Abstract

Hair follicle stem cells (HFSCs) possess fascinating self-renewal capacity and multipotency, which play important roles in mammalian hair growth and skin wound repair. Although HFSCs from other mammalian species have been obtained, the characteristics of ovine HFSCs, as well as the methods to isolate them have not been well addressed. Here, we report an efficient strategy to obtain multipotent ovine HFSCs. Through microdissection and organ culture, we obtained keratinocytes that grew from the bulge area of vibrissa hair follicles, and even abundant keratinocytes were harvested from a single hair follicle. These bulge-derived keratinocytes are highly positive for *Krt15*, *Krt14*, *Tp63*, *Krt19* and *Itga6*; in addition to their strong proliferation abilities *in vitro*, these keratinocytes formed new epidermis, hair follicles and sebaceous glands in skin reconstitution experiments, showing that these are HFSCs from the bulge outer root sheath. Taken together, we developed an efficient *in vitro* system to enrich ovine HFSCs, providing enough HFSCs for the investigations about the ovine hair cycle, aiming to promote wool production in the future.

## 1. Introduction

The mammalian skin is a complicated tissue that contains some elaborate epidermal appendages, for example the hair follicles. A hair follicle is a cylindrical pocket of the epidermis, which is constructed from multiple layers of epithelial cells and extends through the depth of skin [1]. According to systematic observations of the haired mammalian skin, hair follicles undergo continuous cycles of growth, regression, rest and regeneration [2,3,4,5]. Specifically, cell lineage tracing data in transgenic mice have revealed the existence of a fascinating pool of hair follicle stem cells (HFSCs), which are recognized as the outer root sheath (ORS) cells at the bulge [6]. HFSCs provide abundant progenies that are responsible for sustaining the intact structures of hair follicles during continuous hair cycles. Compared to epidermal stem cells, which reside in the basal layer of the interfollicular epidermis (IFE), HFSCs, distinct subpopulations of which reside in different areas of the ORS [1,7], are generally positive for keratin 15 (K15) besides keratin 14 (K14), p63 and integrin α6 [8,9,10,11].

In the past few decades, studies in haired mammalian species, including human, mouse, rat and even canine, have revealed that HFSCs possess extensive self-renewal and multi-differentiation capacity. The first isolated and cultured HFSCs were from rat vibrissa hair follicles [12]. Rat vibrissa hair follicles have bigger, clearer and more homogeneous architectures than other types of hair follicles, greatly facilitating the separation of intact hair follicles and the obtainment of HFSCs [8,11,12,13]. Combined with their highly positive expression of integrin α6 and CD34, HFSCs in *Krt14*-GFP transgenic mice were isolated by fluorescence-activated cell sorting (FACS), and they can form tightly-packed colonies and be passaged for multiple generations *in vitro* [6]. In particular, these cells can generate not only new epidermis, but also hair follicles and sebaceous glands after being grafted into nude mice with dermal cells [6]. A similar study using *Krt15*-EGFP transgenic mice and FACS has validated that mouse HFSCs are able to self-renew and differentiate into multiple cell types *in vitro* and *in vivo* [9]. Due to the technical limitation, *in vitro* acquisition of human HFSCs had not been validated for a long time. However, in 2005, with hair follicles plucked and dissected, human HFSCs proliferated and formed colonies expressing integrin β1 and K15 [14]. Subsequently, the cell surface marker CD200 was used in FACS combined with the exclusion of CD24, CD34, CD71 and CD146 expression to enrich human HFSCs from scalp samples [15]. Distinguished from their homolog, IFE stem cells [16], human HFSCs have not been reported to successfully reconstitute hairy skin in nude mice. In contrast to the species mentioned above, HFSCs in other haired mammals, especially farm animals, are poorly studied, gradually arousing scientific and practical interests. Kobayashi *et al.* dissected and cultured the bulge area of canine hair follicles *in vitro* and obtained highly proliferative keratinocytes, which shared the same marker expression with mouse and human HFSCs [17,18]. *In vitro*, these canine HFSCs formed an IFE structure comprising cell layers that expressed markers similar to the normal epidermis [19].

Although the existence of HFSCs in human, mouse, rat and canine hair follicles has already been demonstrated, there is still a lack of evidence about HFSCs in sheep, an outstanding representative haired livestock. Given the long history of using wool in textile production and the fact that it is still an important economic raw material nowadays, studying the characteristics of ovine HFSCs may help to understand the regulation of the ovine hair cycle and thereby promote wool production.

In the present study, we investigated whether self-renewing and functional HFSCs could be isolated from ovine vibrissa hair follicles and expanded *in vitro*. We firstly microdissected single ovine vibrissa hair follicles without the disruption of enzymatic digestion and cultured the entire mini-organ, ensuring the integrity of the hair follicle structure and the HFSC niche. Cobblestone-like keratinocytes that migrated out of the bulge were subcultured and further examined for the molecular characters and long-term proliferative capacity. Moreover, the multipotency and functionality of these keratinocytes were evaluated in the skin reconstitution assay. In conclusion, our study demonstrated that these ovine bulge-derived keratinocytes have properties of bona fide HFSCs *in vitro*.

## 2. Results

### 2.1. A Feasible Method to Obtain Abundant Ovine Keratinocytes from Single Vibrissa Hair Follicles

**Figure 1:**
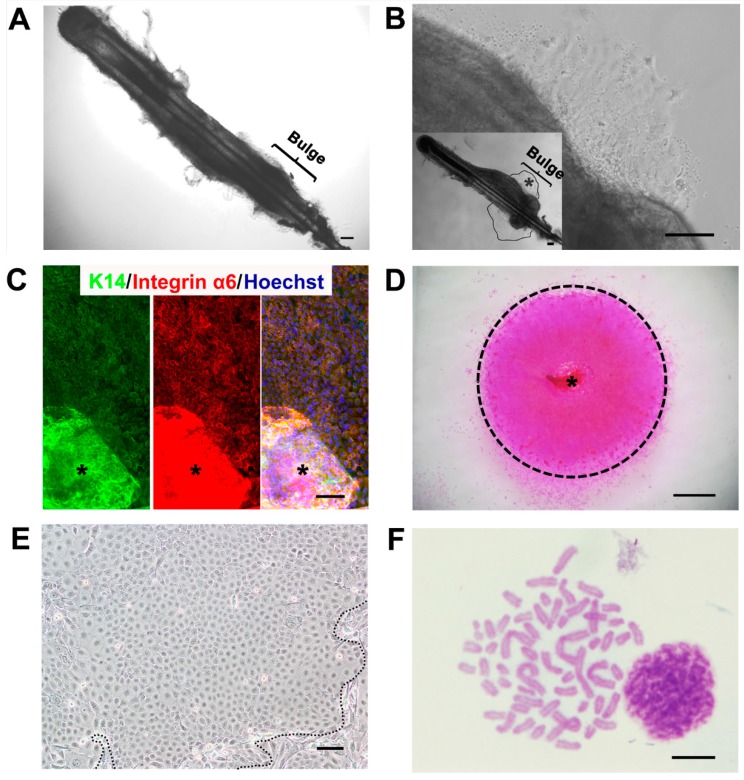
(**A**) A freshly-isolated ovine vibrissa hair follicle. The bugle area is illustrated; (**B**) The cell colony derived from the bulge of the same ovine vibrissa hair follicle after three days of culture. In the bottom-left box, the area within the lines indicates the initial keratinocyte colony. The asterisk indicates the corresponding area shown in (**B**); (**C**) K14 and integrin α6 immunostaining of keratinocytes that migrated from the bulge. The asterisk indicates the bulge; (**D**) A Rhodamine B-stained keratinocyte colony formed around the hair follicle after nine days in culture. The asterisk indicates the vibrissa hair follicle; (**E**) A part of a colony of the ovine bulge-derived keratinocytes and surrounding feeder cells (outside the dashed lines) after passaging; (**F**) Karyotype (57, XX type sex chromosome) of a representative ovine bulge-derived keratinocyte at Passage 15. For (**A**–**C**,**E**), the scale bar is 100 μm; for (**D**), the scale bar is 1 mm; for (**F**), the scale bar is 10 μm.

In order to gain ovine HFSCs, we employed combined methods, including microdissection and organ culture. We previously reported an efficient organ culture system to obtain plenteous HFSCs from limited biopsy, such as a single rat vibrissa hair follicle [11]. Here, following this strategy, we microdissected ovine vibrissa hair follicles under a stereoscope. After the dermal sheath was removed, the bulge was clearly apparent at the distal part (superficial region) of the hair follicle (Figure 1A). The hair follicles were separately cultured in culture dishes with William’s E medium. After three days without disturbance, primary keratinocytes were seen under the microscope growing out from the bulge, which was proven as an important reservoir of mammalian HFSCs [12] (Figure 1B). These keratinocytes formed a packed colony exhibiting a single-layer structure and showed a classical cobblestone-like morphology (Figure 1B). Consistent with their origin, ORS, they were homogeneous for the expression of basal layer markers K14 and integrin α6 (Figure 1C). As shown in Figure 1D, about nine days after the hair follicles were cultured, the colony spread out around the initial hair follicle, with diameters of 5.71 ± 1.07 mm.

Subsequently, the primary keratinocytes were digested gently, seeded into a new dish together with mitomycin C-treated fibroblasts from the ovine ear-rim dermis (which were used as feeder cells) and cultured. Given that keratinocytes had much stronger adherence to the culture dish than fibroblasts, these ovine keratinocytes were easily purified from feeder cells through a two-step process of trypsinization. Although some differentiated cells with larger cell volumes were observed, most keratinocytes displayed the classical cobblestone-like morphology, like other types of epithelial cells (Figure 1E). The differentiated keratinocytes without self-renewal capability cannot undergo the long-term passage as progenitor/stem cells do in our culture and the reported study [6]. Furthermore, karyotype analysis showed that these bulge-derived keratinocytes still had normal chromosome numbers (57) and correct female chromosome-type (XX), even at Passage 15 (P15) (Figure 1F).

The percentage of ovine vibrissa hair follicles that yielded colonies was 94.99% ± 4.41% (Table 1), proving that the combination of microdissection and organ culture was a feasible and efficient strategy to obtain keratinocytes from limited ovine skin materials. The following experiments were performed using bulge-derived keratinocyte populations from only one single vibrissa hair follicle.

**Table 1:** The colony-forming ratio of ovine vibrissa hair follicles.

Group	No. of Hair Follicles Cultured	No. of Hair Follicles that Formed a Colony	Percentage of Yield Ratio
1	12	12	100%
2	15	14	93.33%
3	12	11	91.66%
Average Percentage			94.99%

### 2.2. Expression Profiles of Ovine Bulge-Derived Keratinocytes

**Figure 2:**
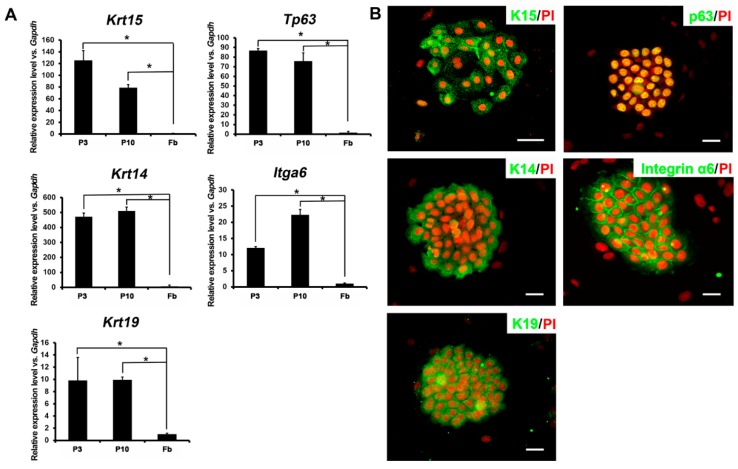
(**A**) qRT-PCR results showing the mRNA expression of *Krt15*, *Tp63*, *Krt14*, *Itga6* and *Krt19* in the ovine bulge-derived keratinocytes at Passage 3 (P3) and P10. Ovine fibroblasts served as a negative control; (**B**) Immunofluorescence staining of K15, p63, K14, integrin α6 and K19 in P6 ovine bulge-derived keratinocyte colonies. Note that the surrounding feeder cells are negative controls for all markers. Fb, fibroblast; scale bar, 50 μm. PI, propidium iodide. * *p* < 0.05.

In order to characterize these ovine bulge-derived keratinocytes, we firstly analyzed the expression of relevant HFSC markers at both the RNA and protein levels. qRT-PCR results showed that P3 and P10 ovine bulge-derived keratinocytes had robust expression of *Krt15*, *Tp63*, *Krt14*, *Itga6* and *Krt19* (Figure 2A). Furthermore, immunofluorescence staining also confirmed the expression of K15, p63, K14, integrin α6 and K19 proteins in P6 ovine bulge-derived keratinocytes, with the surrounding feeder cells as the negative control for every marker (Figure 2B). These keratinocytes showed the typical cytoplasmic distribution of K15 and K14 filaments around the nuclei. The expression of p63 was detected in all of the nuclei within the colony. In addition, integrin α6 expression was enriched at the cell membrane. These results indicate the ORS origins of these keratinocytes.

### 2.3. The Proliferative Capacity of Ovine Bulge-Derived Keratinocytes in Culture

**Figure 3:**
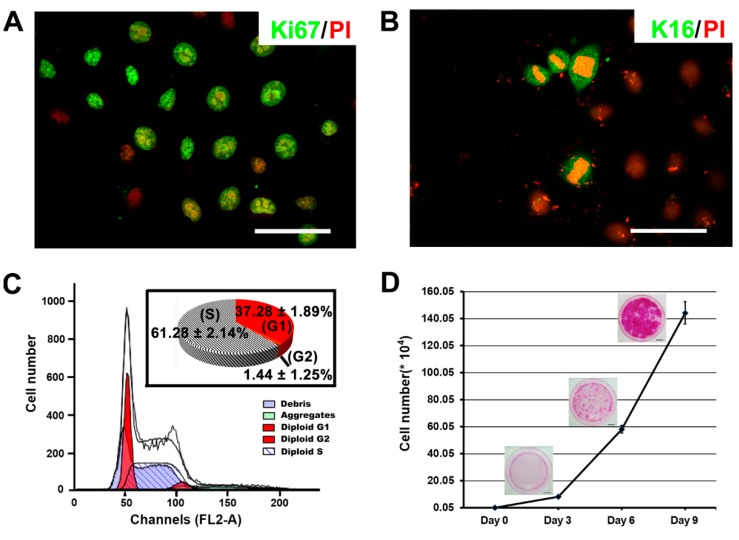
(**A**) Ki67 immunofluorescence staining in P6 ovine bulge-derived keratinocytes three days after passaging; (**B**) K16 immunofluorescence staining in P6 ovine bulge-derived keratinocytes. Note that K16 is present in cells during division, associated with pre-separating, separating and newly-separated chromosomes; (**C**) The cell cycle distribution of the ovine bulge-derived keratinocytes; (**D**) A growth curving showing the proliferation of 500 ovine bulge-derived keratinocytes during nine days of culture, along with the morphology of cell colonies at different time points. For (**A**,**B**), the scale bar is 50 μm; for (**D**), the scale bar is 1 cm.

Stem cells have strong self-renewal capability, which is usually reflected in their robust proliferation *in vitro*. Therefore, we checked the proliferation capacity of these bulge-derived keratinocytes by examining the expression of the proliferation marker Ki67. Most of keratinocytes at P6 (68.94% ± 8.39%) were Ki67 positive on day 3 after passaging, which was considered as the early logarithmic growth phase (Figure 3A). During this phase, there were also some cells showing separating chromosomes, together with the expression of the proliferation-associated keratin K16 (Figure 3B). Besides, the cell cycle distribution analysis performed by FACS illustrated a considerable fraction of cells in the S phase (61.28% ± 2.14%) (Figure 3C). To further quantify the *in vitro* proliferation of the ovine bulge-derived keratinocytes, a cell growth curve assay was conducted. With the seeding density of 500 cells per 6-cm dish, the typical growth curve is shown in Figure 3D. Based on the growth curve, the calculated cell doubling time was about 18 h, and the cell number finally achieved after nine days of culture was about (1.44 ± 0.14) × 10^6^ (Figure 3D). Rhodamine B staining on day 3, 6 and 9 showed continuous expansion of the colonies (Figure 3D). This evidence reveals that the bulge-derived keratinocytes are highly mitotically active in culture, showing typical growth activities of stem cells *in vitro*.

### 2.4. In Vitro Differentiation Capacity of Ovine Bulge-Derived Keratinocytes

**Figure 4:**
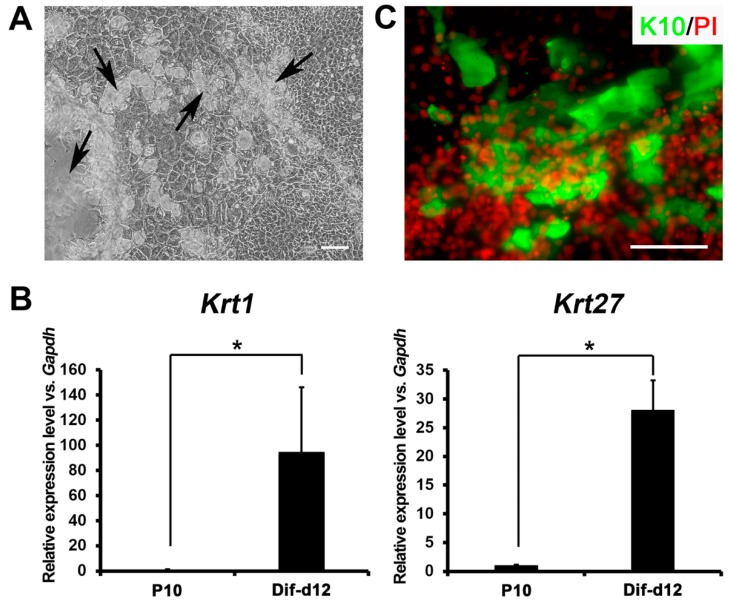
(**A**) Morphology of the differentiated keratinocyte colony after 12 days of confluent culture. The arrows show the multilayer structures formed in the colony; (**B**) qRT-PCR results showing the expression of *Krt1* and *Krt27* in P10 undifferentiated bulge-derived keratinocytes and differentiated keratinocytafter 12 days of confluent culture (Differentiated after day 12 (Dif-d12)); (**C**) Immunostaining of K10 in the differentiated keratinocyte colony after 12 days of confluent culture. Scale bar, 100 μm. * *p* < 0.05.

The *in vitro* differentiation capacity of the ovine bulge-derived keratinocytes into epidermal lineages was assessed. After 12 days of confluent culture, the ovine bulge-derived keratinocytes differentiated spontaneously, and multilayer structures comprised of differentiated cells were found widely distributed in the colonies (Figure 4A). The expression of markers specific for differentiated keratinocytes (*Krt1* and *Krt27*) in the confluent cell mixture were considerably higher than that in P10 undifferentiated bulge-derived keratinocytes (Figure 4B). In addition, the immunofluorescence staining result revealed that the nuclei of these confluent cells piled up, and lots of cells in these multi-layered structures expressed K10, the partner keratin of K1 to form intermediate filaments (Figure 4C). Due to the lack of basic research on sheep dermatology, other possible markers for differentiated ovine keratinocytes were not investigated. These data indicate that these bulge-derived keratinocytes can undergo differentiation in confluent culture *in vitro*, implying their stem cell properties.

### 2.5. Skin Reconstitution with Ovine Bulge-Derived Keratinocytes and Neonatal Dermal Cells

**Figure 5:**
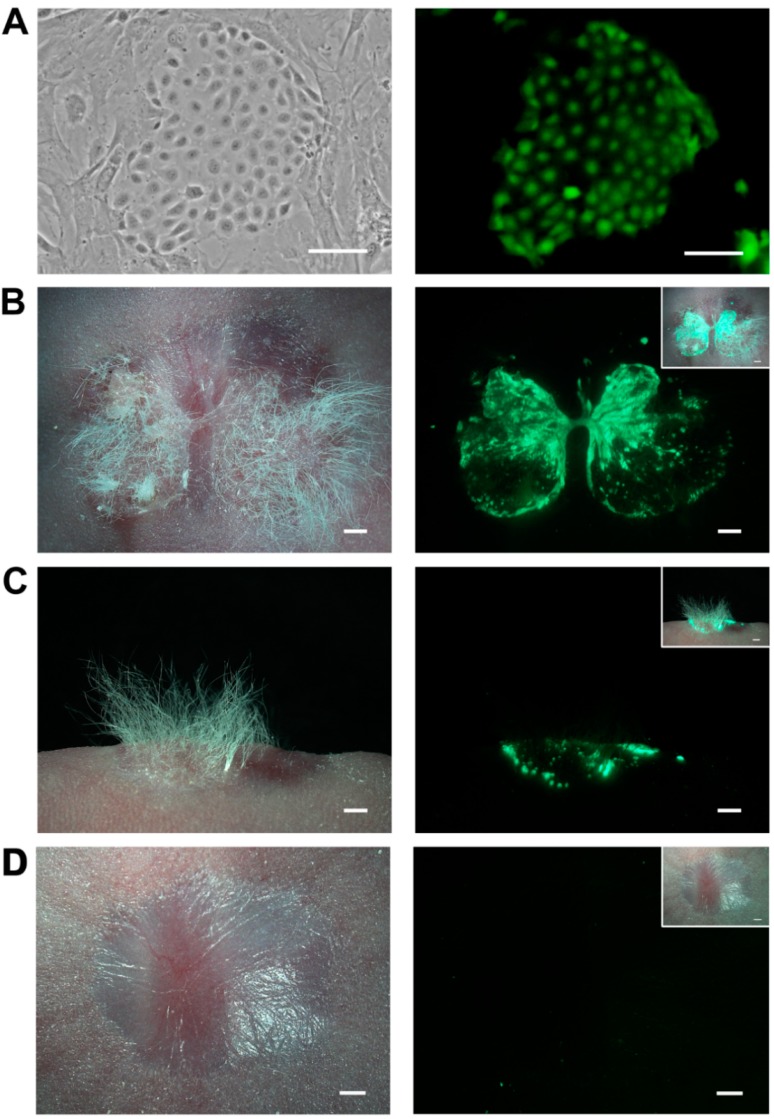
(**A**) A GFP-positive colony of P4 ovine bulge-derived keratinocytes after GFP labeling and FACS. Note the surrounding non-GFP cells are the feeder cells; (**B**) A top view of the graft on the back of the recipient nude mouse four weeks after being grafted with GFP-positive keratinocytes and neonatal CD1 dermal cells; (**C**) A side view of the graft on the back of the recipient nude mouse four weeks after being grafted with GFP-positive keratinocytes and neonatal CD1 dermal cells; (**D**) A top view of the control graft on the back of the nude mouse four weeks after being transplanted with only neonatal CD1 dermal cells. For (**B**–**D**), the upper-right corner shows the merged view of the bright field and the fluorescence. For (**A**), the scale bar is 100 μm; for (**B**–**D**), the scale bar is 1 mm.

It is well known that the more rigid criterion for characterizing HFSC differentiation ability is the successful reconstitution of epidermis and hair follicles in recipient mice after being grafted with dermal cells [20]. Therefore, P3 ovine bulge-derived keratinocytes were labeled with GFP and purified by FACS to facilitate subsequent *in vivo* cell tracing (Figure 5A). After amplification, these GFP-labeled keratinocytes (at P8) were grafted into the excisional full-thickness wound of nude mice, together with neonatal mouse or rat dermal cells. In general, newly-formed hairs were seen in three weeks post grafting (Figure S1). After four weeks of grafting, the hairy skin was more obvious at the wound site grafted with GFP-labeled keratinocytes and dermal cells, showing evident and specific green fluorescence (Figure 5B,C). In contrast, non-haired scars were formed at the wound sites of the control nude mice transplanted with only dermal cells (Figure 5D).

**Figure 6:**
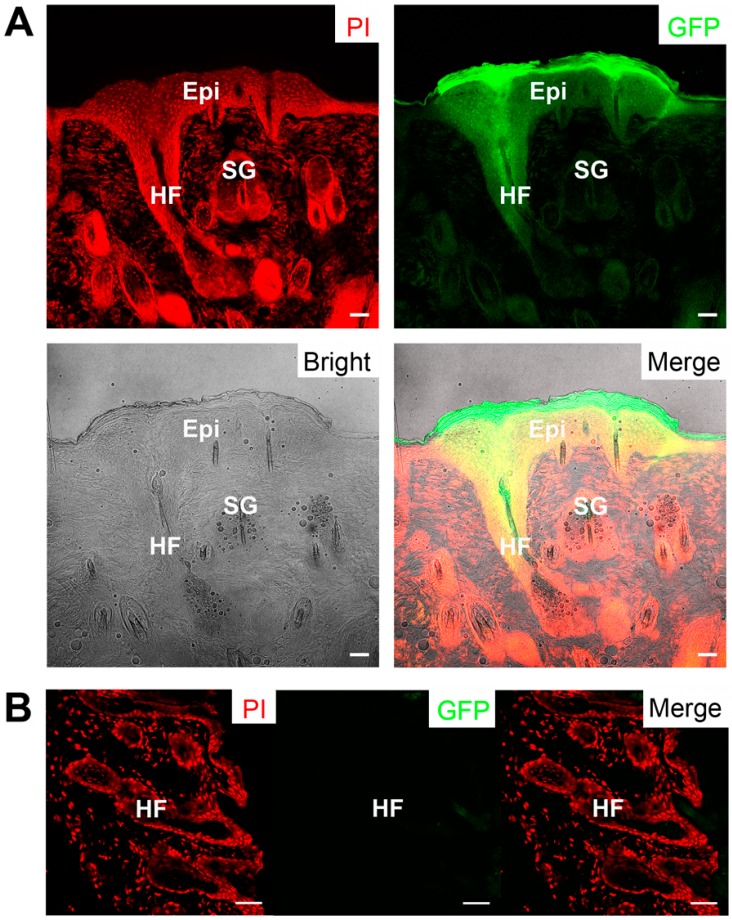
(**A**) The reconstituted hair follicles, multilayer epidermis, and sebaceous glands showing green fluorescence; (**B**) The native skin of the recipient nude mouse as a negative control without green fluorescence. Epi, epidermis; HF, hair follicle; SG, sebaceous gland. Scale bar, 50 μm.

We further examined the structure of the reconstituted haired skin and the newly-formed epidermal appendages. Specifically, the newly-formed hair follicles displayed a strong green fluorescence signal under fluorescent stereomicroscope because of the GFP labeling of transplanted ovine keratinocytes (Figure 6A). Besides, these keratinocytes formed new and green sebaceous glands and IFE (Figure 6A). In contrast, the host skin did not show any GFP signal (Figure 6B). Therefore, the newly-formed hair follicles, sebaceous glands and IFE were generated by the transplanted keratinocytes, demonstrating that these bulge-derived keratinocytes are actually HFSCs.

## 3. Discussion

As an adult stem cell type that is multipotent, easily accessible, non-oncogenic and less limited by ethical issues compared to other pluripotent stem cells [21], HFSCs have great value in fundamental research and promising potentials in the biological engineering. For wool-producing animals, such as sheep, it is of special interest and importance to understand the biology of the hair follicles in order to increase wool production. However, such farm animals have bigger body weights, longer fertility cycles and lower population numbers than laboratory modal animals, largely limiting the application of related research techniques and making it difficult to study stem cell biology in these livestock. To promote the understanding of ovine HFSC behavior, we employed the combination of microdissection and organ culture to isolate and propagate the ovine HFSCs from single vibrissa hair follicles. Intriguingly, these keratinocytes express specific markers as other mammalian HFSCs do and hold robust proliferative capacity *in vitro*. Moreover, new epidermis and related appendages, such as hair follicles and sebaceous glands, can be reconstituted using these keratinocytes and neonatal dermal cells in nude mice. This is the first study to isolate and enrich ovine HFSCs. On the one hand, our findings may provide a stable way to obtain ovine HFSCs to facilitate wool production in the future. On the other hand, this simple system may also provide a kind of adult stem cells as a cell bank to aid physiological and genetic studies in many larger mammalian species, even some rare animals.

A conventional way to isolate HFSCs from mammalian hair follicles is FACS [22,23], which requires the expression of a specific combination of antigen genes at the cell surface and enough amount of skin material. However, for some species, especially human and some rare mammals, the sources of skin biopsies are limited. Another accepted way to obtain HFSCs is the tissue culture of hair follicles [13,24]. Plucking hair follicles out from the skin may cause a loss or disruption of the bulge and the matrix, finally leading to less germinative matrix in the culture [25]. Some researchers attempted to cut dissected hair follicles into two or three parts vertically and cultured these parts separately [13,15,18,24]. However, once the hair follicle is cut into pieces, the niche is damaged, and the HFSCs lose some of the important signals from the niche. Unlike the studies above, we used the combination of microdissection and intact organ culture of single ovine hair follicles in the present study, thereby reserving the natural and physiological HFSC niche to the greatest extent and facilitating the *in vitro* propagation of HFSCs without losing their stem cell activities. More importantly, these keratinocytes we obtained have a definite origin, which is the ORS of hair follicles, rather than the basal layers of epidermis. Meanwhile, the high HFSC yield ratio from the ovine vibrissa hair follicle bulges in the present study demonstrated the suitability and efficiency of the combination of microdissection and organ culture for the acquisition of HFSCs from livestock.

However, for the combination of microdissection and organ culture, there are still defects regarding the purity and homogeneousness of isolated cells. In fact, for mouse, there are different kinds of HFSCs at different locations of the ORS of hair follicles, such as isthmus HFSCs, upper and lower bulge HFSCs, and so on [1,7]. In our initial cultures, there were residual dermal cells that can be eliminated by the two-step trypsinization and some keratinocytes that were deemed as transient amplifying cells that would differentiate after short-term passages. Only the keratinocytes from the ORS, which are homogeneous for the expression of basal layer marker K14 and integrin α6 (Figure 1C), have the ability of self-renewal. After long-term *in vitro* culture and passage, these keratinocytes, which still actively proliferate, are assumed as only a mixture of upper and lower bulge HFSCs. Markers distinguishing the upper and lower bulge HFSCs in sheep need to be developed in the future.

Some studies have led to the view that HFSCs from human, mouse, rat and even canine share a typical gene-expression profile, such as the abundant expression of basal layer cell markers K14 [26], p63 [27,28] and integrin α6 [29] and HFSC markers K15 [30], K19 [31] and LGR5/6 [32,33]. Nonetheless, certain genes were highly expressed in hair follicles in some species, but not in others, for example CD200 (human and canine) [15,18] and CD34 (mouse and rat) [13,22]. According to our data, ovine HFSCs from single vibrissa hair follicles also express these basal layer markers mentioned above, just like in other mammalian HFSCs. Notably, K15 expression has been reported as a stem cell marker specific for the bulge area in mouse hair follicles [30,34]. However, some findings have demonstrated the diverse levels of regulation and distribution of K15 expression in other mammalian skin [35,36,37]. Whitbread and Powell found that in sheep skin, K15 exhibits a basal expression pattern in the ORS of hair follicles, IFE and sebaceous glands, but not in the bulge [38]. However, using immunofluorescence staining on both ovine upper-lip skin (Figure S2A) and posterior neck skin (Figure S2B), we found that K15 was expressed in the basal layer of the whole ORS (including that of the bulge), IFE and sebaceous glands. Furthermore, bulge-derived keratinocytes in culture expressed K15 (Figure 2A,B). Although K15 cannot be used as a bulge stem cell marker in ovine hair follicles, we have employed other assays, such as the detections of proliferation and differentiation capacities *in vitro* (Figure 3 and Figure 4) and the skin reconstitution assay *in vivo* (Figure 5 and Figure 6), to identify the ovine HFSCs that we obtained and further demonstrate their stem cell properties. These results together demonstrated that these keratinocytes are HFSCs. Microarray comparison of ovine HFSCs and IFE stem cells would help further characterize the unique gene expression profile of ovine HFSCs, and works are still needed to find specific ovine HFSC markers.

*In vivo*, bulge stem cells (HFSCs) can contribute to epidermis, hair follicles and sebaceous glands, three epidermal lineages of skin [6,9,11,24]. Therefore, *in vitro*, the capability of HFSCs to differentiate into epidermal lineages provides the possibility that HFSCs can be a potential accessible, autologous source of adult stem cells for regenerative medicine and bioengineering [39]. The skin reconstitution assay in nude mice is a classical approach to investigate the multipotency of keratinocyte progenitors [9,40,41]. In the present study, GFP labeling enabled the specific tracking of the progenies of grafted HFSCs. According to our observation, GFP-positive hair follicles were observed in the reconstituted skin, which is evidence for the differentiation ability of ovine HFSCs that we obtained. Moreover, P8 GFP-expressing ovine HFSCs can be reorganized and differentiate into epidermal lineages in a skin reconstitution assay, showing that these HFSCs retain their multipotency after long-term proliferation.

There are a series of assays that are required to fully verify the HFSC identity. The expression of a group of marker genes and the ability of cells to differentiate into lineages of hair follicles, even epidermis, are necessary criteria to attest to the founder cells of hair follicles. Currently, lineage-tracing techniques, which can be used to track the progeny of a group of cells, even of a single cell, are accepted as the most rigid strategy to identify endogenous adult stem cells *in vivo*, including HFSCs [42,43,44]. In mouse hair follicles, using lineage-tracing techniques, several subsets of HFSCs have been discovered, locating at different regions and exerting distinct functions during hair follicle and epidermal homeostasis [1,7,33,45]. However, limited by the traits of livestock, which have been mentioned above, we can only adopt some feasible methods to study the characteristics of ovine HFSCs we obtained, such as the marker gene expression and tissue formation after grafting. Although, without the lineage tracing assay, which we cannot implement in sheep, according to all of the assays we performed, our ovine HFSCs showed representative *in vitro* activities and capacities of bona fide HFSCs from other species, such as mouse and rat.

In conclusion, the present study highlights the attainability of abundant multipotent proliferative ovine HFSCs from single ovine vibrissae with a simple and efficient method. The *in vitro* system we established here holds great promise to investigate the hair cycle and the hair follicle development in sheep, possibly promoting wool production and, maybe, providing a potential alternative bioreactor for drug tests in the agricultural industry. Furthermore, our study, perhaps, makes it possible to obtain another highly proliferative stem cells, besides ovine-induced pluripotent stem cells [46], for nuclear transfer or individual-specific data collection in some rare haired mammals, such as Tibetan antelope, in the future.

## 4. Experimental Section

### 4.1. Animals

Adult Ujimqin sheep (provided by the College of Animal Science and Technology, Beijing University of Agriculture, Beijing, China), CD1 mice and Wistar rats (provided by Vital River Laboratory Animal Center, Beijing, China) were euthanized in accordance with the Guideline of the Ethics Committee of the Institute of Zoology, Chinese Academy of Sciences (CAS). Nude mice (nu/nu, also provided by Vital River Laboratory Animal Center) were raised under specific-pathogen-free (SPF) conditions with a constant photoperiod (12 hour light:12 hour dark) and free access to water and food. All animal experiments were performed (under project identification code, the Strategic Priority Research Program of the CAS XDA01010202, 201101-201512) with the consent of the Ethics Committee of the Institute of Zoology, CAS. All events were executed in accordance with the Guide for the Care and Use of Laboratory Animals issued by the Institute of Zoology, CAS.

### 4.2. Microdissection

Ovine vibrissa hair follicles were microdissected as described before [11]. Briefly, after the blunt dissection of ovine upper-lip skin, with forceps and 25-G syringe tips (Becton-Dickenson, Franklin Lakes, NJ, USA), the single vibrissa hair follicles were carefully dissected, and dermal sheaths covering hair follicles were carefully removed under a stereoscope. Then, one intact hair follicle was cultured at the bottom of a well of a six-well plate (Corning, Corning, NY, USA) at 37 °C/5% CO_2_ in William’s E medium (Gibco, Carlsbad, CA, USA) containing 15% fetal bovine serum (FBS) (Hyclone, Logan, UT, USA), penicillin-streptomycin, EGF, insulin, hydrocortisone, and so on (all from Sigma, St. Louis, MO, USA) [22]. The experiments of microdissection and organ culture were repeated three times, with more than ten hair follicles dissected and cultured each time. After cells grew out, the growth medium was changed every other day.

### 4.3. Cell Culture and Passaging

Ovine fibroblasts were derived from ear-rim dermis. Briefly, ovine ear-rim skin was degermed by multiple washes with saline. The epidermis and dermis were separated with 5 mg/mL dispase (Gibco, Carlsbad, CA, USA), and the dermis was further digested with 10 mg/mL type IV collagenase (Gibco, Carlsbad, CA, USA) into single cells, which were seeded at a density of 2 × 10^6^ per 10-cm dish and cultured in Dulbecco’s Modified Eagle’s Medium (DMEM) (Gibco, Carlsbad, CA, USA) supplemented with 10% FBS and penicillin-streptomycin. For preparing feeder cells, P3 dermal fibroblasts were pre-treated with 10 μg/mL mitomycin C (Sigma, St. Louis, MO, USA).

As previously described [11], we used a two-step trypsinization procedure to purify and passage the ovine bulge-derived keratinocytes. Briefly, feeder cells were first discarded after digested with 0.05% trypsin-EDTA (Gibco, Carlsbad, CA, USA) at room temperature. Subsequently, for the digestion of the bugle-derived keratinocytes, we applied 0.05% trypsin-EDTA at 37 °C. The single-cell suspension of keratinocytes was re-plated with feeder cells. The growth medium was changed every other day.

To evaluate the cell proliferation capacity, ovine bugle-derived keratinocytes were seeded into 6-cm dishes at a density of 500 cells/dish. Cells were harvested every three days, and the cell numbers were counted. Each time point had a triplication, and the cell numbers were shown as means ± SEM. To calculate the cell doubling time of HFSCs, the following formula was applied:

DT = t × [lg2/(lgNt - lgNo)]
(1)

DT indicates the doubling time; t indicates the culture hours; Nt indicates the final cell numbers; No indicates the initial cell numbers. To visualize the keratinocyte colonies, colonies were fixed with 4% paraformaldehyde (PFA), stained with 1% Rhodamine B (both from Sigma, St. Louis, MO, USA), and then, pictures were taken with a Canon G12 camera (Canon, Ohta-ku, Tokyo, Japan).

### 4.4. Fluorescence-Activated Cell Sorting (FACS)

To analyze the cell cycle distribution, bulge-derived keratinocytes were collected and washed twice with phosphate-buffered saline (PBS). Then, the cells were re-suspended and fixed in 70% ethanol at 4 °C overnight. Before being analyzed with a BD Calibur flow cytometer (Becton-Dickenson, Franklin Lakes, NJ, USA), cells were collected and washed with PBS and then stained with 50 μg/mL propidium iodide (PI) in PBS complemented with 100 μg/mL RNase A and 0.2% Triton X-100 (all from Sigma, St. Louis, MO, USA) for 30 min at 4 °C. In total, 3 cell cultures were used for cell cycle analysis, and 10^4^ cells were analyzed for each sample.

### 4.5. Karyotype Analysis

Karyotype analysis was done according to standard murine chromosome analysis protocols [47]. In brief, cells were pretreated with 0.02 μg/mL colchicine for 3 h and then collected. The cell pellet was resuspended with 0.075 M KCl (Sigma, St. Louis, MO, USA) and was incubated at 37 °C for 20 min. Then, cells were fixed with methanol/acetic acid (3:1, all from Sigma, St. Louis, MO, USA), and the cell suspension was dripped onto the slides and stained with Giemsa (Beijing Dingguo Changsheng Biotechnology Co., Ltd., Beijing, China). The stained slides were observed with a Nikon Fluorescence Microscope E80i (Nikon, Tokyo, Japan).

### 4.6. RNA Purification, Reverse Transcription and qRT-PCR

P3 and P10 ovine bulge-derived keratinocytes, as well as ovine ear-rim dermal fibroblasts, which were used as a negative control, were harvested, and total RNAs were extracted using TRIzol reagent (Invitrogen, Carlsbad, CA, USA) according to the manufacturer’s protocol. After the removal of genomic DNA with RQ1 RNase-Free DNase (Promega, Madison, WI, USA), the reverse transcription was performed with M-MuLV reverse transcriptase (New England Biolabs, Beverly, MA, USA).

qRT-PCR reactions were performed in 20-μL volumes using GoTaq qRT-PCR master mix (Promega, Madison, WI, USA) on a Roche LightCycler480 system (Roche Applied Science, Indianapolis, IN, USA). The qRT-PCR procedure was as follows: 94 °C for 2 min, 40 cycles of 95 °C for 15 s, 55 °C for 15 s and 68 °C for 25 s.

The primers used are listed in Table S1. Gene expression relative to *Gapdh* was calculated using the ΔΔ*C*_t_ method with efficiency correction [48].

### 4.7. Immunofluorescence

P6 ovine bulge-derived keratinocytes were seeded on the coverslips for about three days to form colonies. Then, the cells were fixed in 4% PFA and rinsed with PBS three times. Non-specific labeling was blocked by pre-incubation with 5% rabbit serum (both from Sigma) together with 0.1% Triton-X 100 in PBS at 37 °C for 1 h. Primary antibodies were applied in PBS with 1% bovine serum at 4 °C overnight. Corresponding secondary antibodies (Zhongshan Goldenbridge Biotechnology Co., Ltd., Beijing, China) were employed respectively at 37 °C for 1 h. The nuclei were counterstained with PI or Hoechst 33342 (both form Sigma, St. Louis, MO, USA). A Zeiss LSM710 confocal light microscope (Carl Zeiss, Jenna, Germany) was used for the observation and imaging.

The primary antibodies are listed in Table S2.

### 4.8. GFP Lentivirus Production and Keratinocyte Infection and Sorting

The 2K7-GFP lentiviral vectors for infection were packaged in HEK-293FT cells (Invitrogen, Carlsbad, CA, USA) using Fugene HD transfection reagent (Promega, Madison, WI, USA) with the ViraPowe™ Lentivirus Packaging System (Invitrogen, Carlsbad, CA, USA) as previously described [49]. To infect the bulge-derived keratinocytes, approximately 10^5^ P3 bulge-derived keratinocytes were incubated with growth medium containing concentrated lentiviruses and 8 μg/mL polybrene (Sigma, St. Louis, MO, USA) for 24 h. Virus-containing growth medium was replaced with fresh medium the next day. Upon confluence, these GPF-labeled bulge-derived keratinocytes were trypsinized into single cell suspension and sorted with a BD Aria flow cytometer (Becton-Dickenson, Franklin Lakes, NJ, USA). Cells with strong green fluorescence were collected and cultured with the previously-described method.

### 4.9. Skin Reconstitution Assay

The skin reconstitution method used in this study is modified from a previous report [41]. Briefly, the neonatal Wistar rat or CD1 mouse dermal cells were prepared as previously described [50]. For each recipient nude mouse, 1.8 × 10^7^ dermal cells in 75 μL DMEM and 3.6 × 10^6^ GPF-labeled bulge-derived keratinocytes (P8) in 75 μL DMEM were mixed well with 50 μL Matrigel (Becton-Dickenson, Franklin Lakes, NJ, USA). A circular and full-thickness cutaneous wound was made in the back of an anesthetized nude mouse (about 7 weeks old), and the mixture was grafted onto the wound area. The mice were euthanized four weeks after grafting. The skin samples at the graft sites were dissected and then cryosectioned into 40 μm-thick slices using the CM1950 platform (Leica Instruments, Nussloch, Germany). After being fixed in 4% PFA, slides were stained with PI for nuclear identification and were subsequently examined and photographed under the Zeiss LSM780 confocal light microscope (Carl Zeiss). The reconstitution assay was repeated more than three times, each time with more than three mice grafted with the cell mixture and one mouse grafted only with dermal cells as a negative control.

### 4.10. Statistical Analysis

At least 3 independent experiments were used for statistical analysis. Differences were analyzed using one-way ANOVA and Bonferroni *post hoc* test with SPSS 16.0. Data were presented as the means ± SEM. Differences were considered significant if the *p*-value <0.05.

## Supplementary Materials

Supplementary materials can be found at http://www.mdpi.com/1422-0067/16/08/17779/s1.
