# Supplementary material for: Ovine Hair Follicle Stem Cells Derived from Single Vibrissae Reconstitute Haired Skin
Source: Int J Mol Sci. 2015 Aug 3;16(8):17779–97. doi: 10.3390/ijms160817779 (PMC4581221; doi:10.3390/ijms160817779)
Supplement: Supplementary file 1 [file ijms-16-17779-s001.pdf]

Supplementary Information

Table S1. Primer sequences used for qRT-PCR.

Gene	Primers
Krt15	Forward: GCAAGGTCCGCATCAAC<br>Reverse: AGAAACGAAGGACCCCTTG
Tp63	Forward: CTTCCTCAGCACACGATTGA<br>Reverse: GCTGCTGAGGGTTGATAAGC
Krt14	Forward: CACGAGGAGGAGATGAAAGC<br>Reverse: CTCCGTCTTGCTGAAGAACC
Itga6	Forward: TAGTTGGTGAGCAAGCGATG<br>Reverse: CCATTTGCCGTTGCTAATTT
Krt19	Forward: TGAGCGTGGAGACTGACATC<br>Reverse: TGATTTCCTCCTCGTGGTTC
Krt1	Forward: GCAACATGGAGACAGCTTGA<br>Reverse: TCCTTAATGGCGTTCTCACC
Krt27	Forward: CCATCTAAAGCCACCGTG<br>Reverse: CACCCTCTGTTCACTCTTCAT
Gapdh	Forward: GTCCGTTGTGGATCTGACCT<br>Reverse: CGGGAGATTCTCAGTGTGGT

Table S2. Antibodies used for immunofluorescence staining.

Primary Antibody	Vendor and Catalog	Dilution
K15	Santa Cruz, sc-69554	1:100
p63	Santa Cruz, sc-8431	1:100
K14	Millipore, CBL197	1:100
Integrin α6	Biolegend, 313602	1:100
K19	Zhongshan Goldbridge, ZM-0074	1:100
Ki67	eBioscience, 14-5698	1:200
K16	ThermoFisher, MA5-13730	1:100

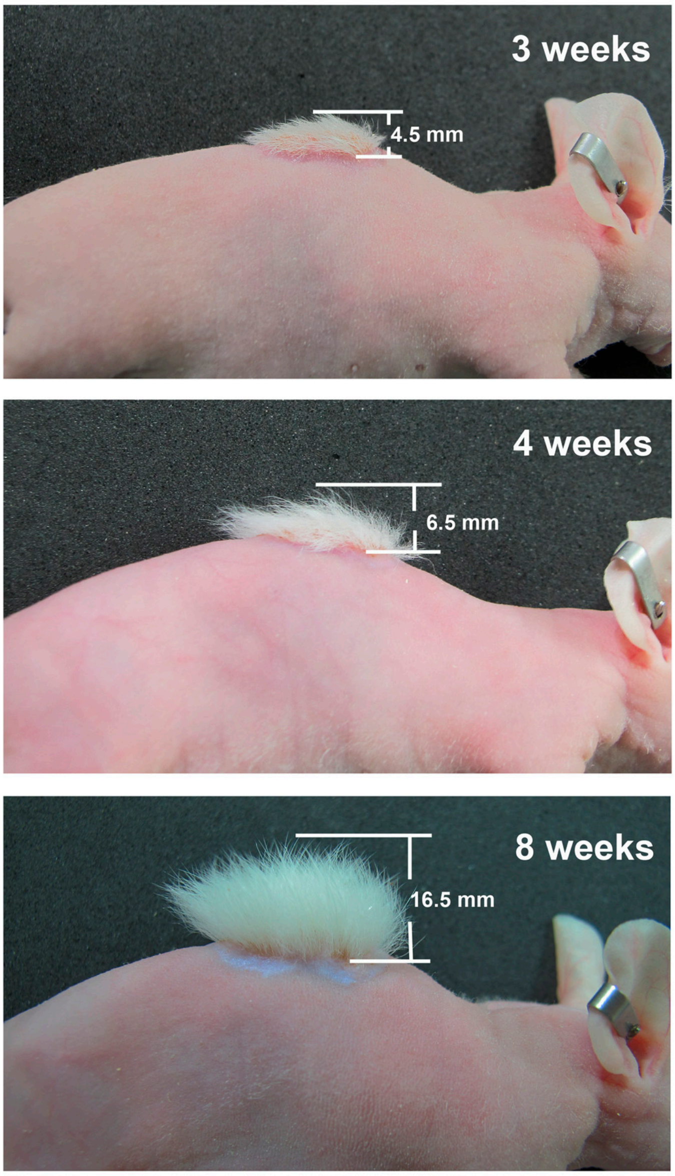

Figure S1. Hair growth of the hair follicles reconstituted by the ovine bulge-derived keratinocytes and neonatal rat dermal cells.

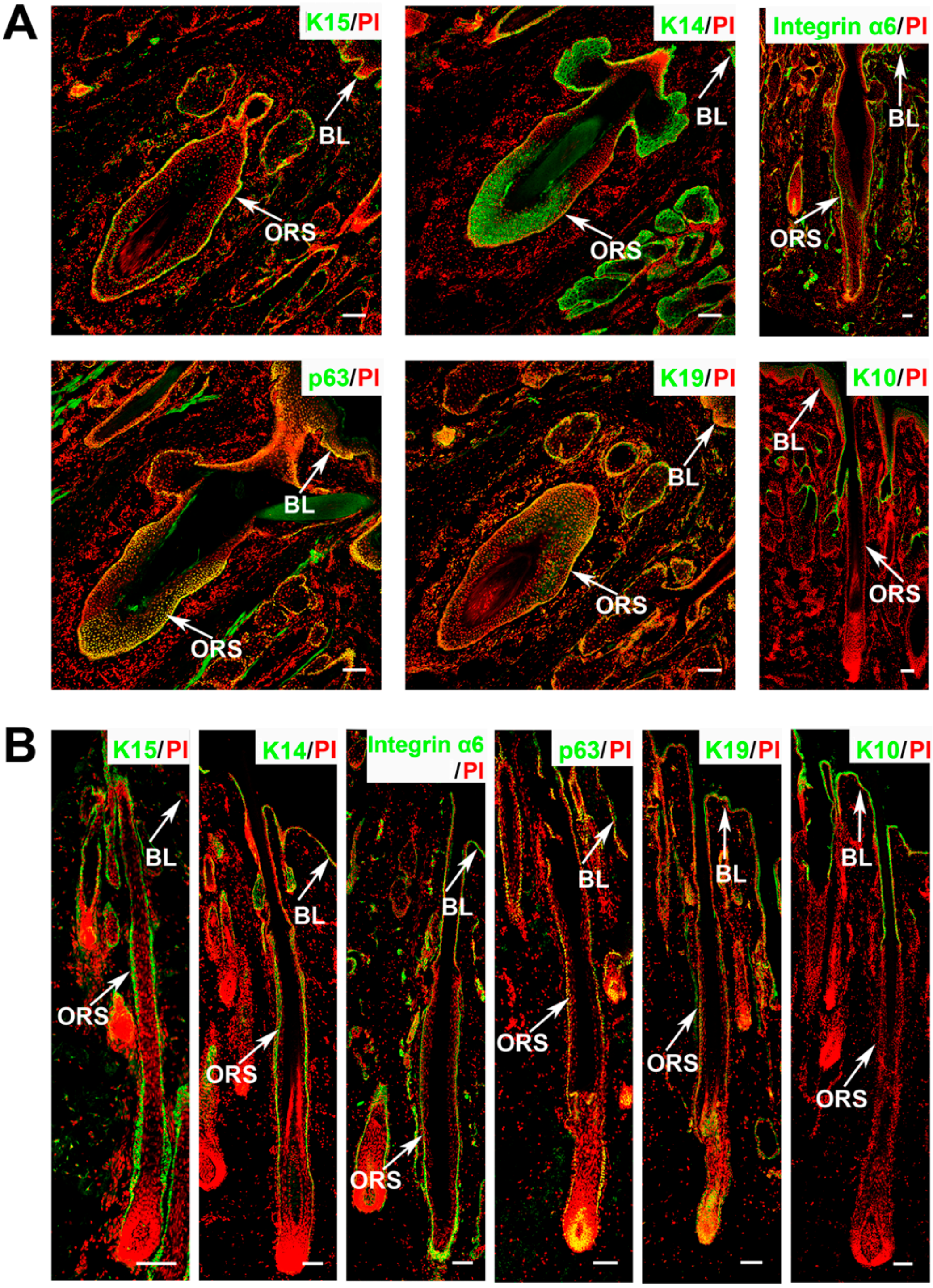

Figure S2. The expression of markers in ovine vibrissa skin and posterior neck skin. (**A**) Immunostaining of K15, K14, Integrin α6, p63, K19 and K10 in the ovine upper-lip skin including the vibrissa hair follicles; (**B**) Immunostaining of K15, K14, Integrin α6, p63, K19 and K10 in the ovine posterior neck skin. BL, basal layer; ORS, outer root sheath. Scale bar, 50 μm.
